# Cardiovascular Burden of Narcolepsy Disease (CV-BOND): a real-world evidence study

**DOI:** 10.1093/sleep/zsad161

**Published:** 2023-06-12

**Authors:** Rami H Ben-Joseph, Ragy Saad, Jed Black, Elizabeth C Dabrowski, Ben Taylor, Sophia Gallucci, Virend K Somers

**Affiliations:** Jazz Pharmaceuticals, Palo Alto, CA, USA; Jazz Pharmaceuticals, Palo Alto, CA, USA; Jazz Pharmaceuticals, Palo Alto, CA, USA; Stanford University Center for Sleep Sciences and Medicine, Palo Alto, CA, USA; Aetion, Inc., New York, NY, USA; Aetion, Inc., New York, NY, USA; Aetion, Inc., New York, NY, USA; Department of Cardiovascular Medicine, Mayo Clinic, Rochester, MN, USA

**Keywords:** narcolepsy, comorbidities, cardiovascular, stroke

## Abstract

**Study Objectives:**

Narcolepsy is associated with cardiovascular risk factors; however, the risk of new-onset cardiovascular events in this population is unknown. This real-world study evaluated the excess risk of new-onset cardiovascular events in U.S. adults with narcolepsy.

**Methods:**

A retrospective cohort study using IBM MarketScan administrative claims data (2014–2019) was conducted. A narcolepsy cohort, comprising adults (≥18 years) with at least two outpatient claims containing a narcolepsy diagnosis, of which at least one was non-diagnostic, was matched to a non-narcolepsy control cohort (1:3) based on cohort entry date, age, sex, geographic region, and insurance type. The relative risk of new-onset cardiovascular events was estimated using a multivariable Cox proportional hazards model to compute adjusted hazard ratios (HRs) and 95% confidence intervals (CIs).

**Results:**

The narcolepsy and matched non-narcolepsy control cohorts included 12 816 and 38 441 individuals, respectively. At baseline, cohort demographics were generally similar; however, patients with narcolepsy had more comorbidities. In adjusted analyses, the risk of new-onset cardiovascular events was higher in the narcolepsy cohort compared with the control cohort: any stroke (HR [95% CI], 1.71 [1.24, 2.34]); heart failure (1.35 [1.03, 1.76]); ischemic stroke (1.67 [1.19, 2.34]); major adverse cardiac event (1.45 [1.20, 1.74]); grouped instances of stroke, atrial fibrillation, or edema (1.48 [1.25, 1.74]); and cardiovascular disease (1.30 [1.08, 1.56]).

**Conclusion:**

Individuals with narcolepsy are at increased risk of new-onset cardiovascular events compared with individuals without narcolepsy. Physicians should consider cardiovascular risk in patients with narcolepsy when weighing treatment options.

## Graphical Abstract

**Graphical Abstract:**
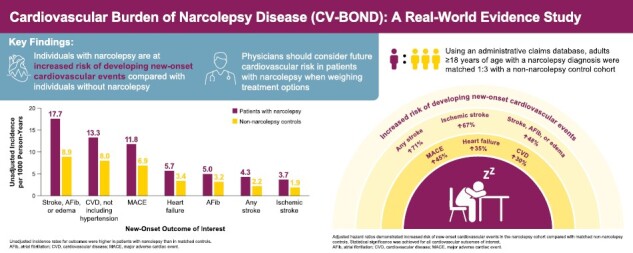

**Statement of Significance**

This study builds on the current understanding of the association between cardiovascular health and sleep with the novel finding that new-onset cardiovascular events occur at a higher rate in people with narcolepsy than in those without narcolepsy. In this observational, retrospective cohort study of a claims database, compared with matched non-narcolepsy controls, people with narcolepsy had a greater risk of new-onset cardiovascular events, including any stroke; heart failure; ischemic stroke; major adverse cardiac event; grouped instances of stroke, atrial fibrillation, or edema; and cardiovascular disease.

## Introduction

Narcolepsy is a rare central disorder of hypersomnolence characterized by a pentad of symptoms [1]. Excessive daytime sleepiness occurs in all patients, whereas cataplexy, disrupted nighttime sleep, hypnagogic and hypnopompic hallucinations, and sleep paralysis occur in some patients. Two subtypes of narcolepsy are recognized, denoted as narcolepsy subtype 1 (NT1) and subtype 2 (NT2). In NT1, either cataplexy is present or cerebrospinal fluid (CSF) hypocretin-1 levels are reduced. In NT2, cataplexy is absent and CSF hypocretin-1 levels are >110 pg/mL, or more than one-third of normal levels. Narcolepsy is associated with functional impairment in multiple domains, including academic, professional, and social, as well as reduced quality of life [2–4]. Although diagnosis is often delayed [5, 6], symptom onset typically occurs in adolescence or young adulthood, resulting in long-term treatment of nighttime and daytime symptoms of most patients [7].

Treatments commonly prescribed for narcolepsy carry cardiovascular risks and related precautions [8]. For example, a retrospective analysis showed a greater proportion of patients taking modafinil required new or increased use of antihypertensive medications (2.4%) compared with placebo (0.7%) [9]. Consequently, increased monitoring is recommended for patients with cardiovascular disease taking this medication [10]. People with NT1 treated with stimulants exhibited increased heart rate and blood pressure, compared with untreated patients [11]. These effects were magnified when stimulants were taken concurrently with anticataplectic medications [11]. The American Academy of Sleep Medicine (AASM) strongly recommends sodium oxybate (SXB) for the treatment of multiple narcolepsy symptoms [12]. However, at a total daily dose of 9 g/night, SXB alone (containing ≈1640 mg sodium) contributes approximately 71% of the 2300-mg daily adult upper limit recommended by authoritative bodies (recommended limits for children are lower than adults) [13, 14] and more sodium than optimal intake (<1500 mg) recommended by the American College of Cardiology and American Heart Association Guidelines on the Primary Prevention of Cardiovascular Disease [15]. Excess dietary sodium intake is additive to the sodium content of SXB [16]. A large national study in the United States reported that 89% of adults exceeded dietary sodium intake of 2300 mg/day and 92% to 94% of children (2–18 years of age) consumed excess sodium [17]. The National Academies of Sciences, Engineering, and Medicine concluded that reducing sodium intake by 1000 mg/day among individuals with intakes above the upper limit of 2300 mg/day can reduce the risk of cardiovascular disease and hypertension by 27% and 20%, respectively [14]. Therefore, the U.S. Food and Drug Administration advises clinicians to closely monitor patients receiving SXB who have heart failure, hypertension, or impaired renal function [18]. Similar precautions are advised for newer therapies, such as pitolisant (Wakix) and solriamfetol (Sunosi), which are associated with QT interval prolongation and increases in blood pressure and heart rate, respectively [19, 20].

Although elevated cardiovascular risk in people with narcolepsy has been reported in previous studies, those associations were cross-sectional, and thus it was not certain that narcolepsy preceded the onset of cardiovascular comorbidities [8, 21–23]. Furthermore, those previous studies could not disentangle whether the increased prevalence of cardiovascular events was inherent to narcolepsy or a result of associated comorbidities and risk factors (e.g. disrupted nocturnal blood pressure dipping) [24]. Thus, the causal association between narcolepsy and the risk of new-onset cardiovascular events, independent of other factors, has not been conclusively demonstrated. Improved understanding of this risk is essential for optimizing treatment, considering the negative cardiovascular effects of narcolepsy medications and the need for long-term therapy. The objective of this study was to estimate the excess risk of new-onset cardiovascular events in adult patients with narcolepsy in the United States.

## Methods

### Study design

This was a retrospective cohort study using administrative claims from the IBM MarketScan (now Merative MarketScan) database. MarketScan contains de-identified patient-level data, including medical and drug data, contributed by large employers, managed care organizations, hospitals, electronic medical record providers, Medicare, and Medicaid [25]. The database is nationally representative of Americans with employer-provided health insurance [25]. This analysis included commercial and Medicare insurance claims of adult patients available in the database for the study period of January 1, 2014 to June 30, 2019.

### Study population

Eligible patients were 18 years of age or older with medical and prescription coverage at cohort entry. Patients were excluded if they did not have at least 6 months (represented as 180 days) of continuous enrollment prior to cohort entry. “Enrollment” describes the time period during which patients are enrolled in their health plan, as captured in the MarketScan database. “Continuous enrollment” is defined as a period of enrollment with a maximum 30-day allowable gap in health plan coverage.

The narcolepsy cohort was defined by the earliest available two outpatient claims containing a diagnosis of NT1 (*International Classification of Diseases, Ninth Revision, Clinical Modification* [ICD-9-CM]: 347.01, 347.11; *International Classification of Diseases, Tenth Revision, Clinical Modification* [ICD-10-CM]: G47.411, G47.421) or NT2 (ICD-9-CM: 347.00, 347.10; ICD-10-CM: G47.419, G47.429) on separate visits no more than 6 months apart, of which at least one claim was non-diagnostic (i.e. at least one claim must not have had an indication of multiple sleep latency test, polysomnography [PSG], or other diagnostic testing; Supplementary Table S1) [26]. The earliest date on which these criteria were met (i.e. on the second claim) served as the date of cohort entry. Patients with medical claims for both NT1 and NT2 were assumed to have NT1 because cataplexy does not resolve [27].

Non-narcolepsy controls were matched to patients with narcolepsy in a 3:1 ratio by calendar date of cohort entry, age, sex, U.S. geographic region (Northeast, North Central, South, West, and unknown), and insurance type (commercial or Medicare) to form a comparable matched sample of the general population of U.S. adults without a diagnosis of narcolepsy prior to cohort entry.

### Outcomes

The outcomes that were assessed include any stroke; atrial fibrillation; heart failure; ischemic stroke; major adverse cardiac event (MACE; defined as grouped instances of myocardial infarction, ischemic stroke, heart failure, acute coronary syndrome, coronary artery bypass grafting, or percutaneous coronary intervention) [28]; myocardial infarction; grouped instances of stroke, atrial fibrillation, or edema; and cardiovascular disease (CVD; grouped instances of stroke, atrial fibrillation, heart failure, and myocardial infarction). Outcomes were defined using ICD-9-CM and ICD-10-CM codes (Supplementary Table S2). Each outcome of interest was classified as a new-onset cardiovascular event if the first evidence of the event in the database occurred during the follow-up period, which began 1 day after cohort entry. Patients who had a history of the outcome of interest in the 6 months prior to cohort entry were excluded from the analysis of that outcome; patients who experienced a first occurrence of an outcome of interest after cohort entry were likewise censored for analysis of later such outcomes. Each remained eligible for inclusion in analyses of other outcomes of interest.

### Analyses

**Figure 1.**
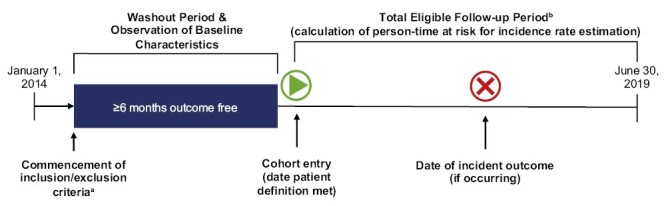
Study design. ^a^Inclusion criteria included ≥6 months of continuous enrollment. ^b^Patients were censored at discontinuation of insurance coverage, at the end of the study period (for those continuously enrolled), or at first qualifying diagnosis for the outcome of interest.

Patients were followed until the earliest day of insurance coverage discontinuation, occurrence of the outcome, or end of the data collection period (Figure 1). Differences in baseline characteristics between the narcolepsy and non-narcolepsy cohorts were evaluated; for binary variables, this was based on a two-sample comparison of independent proportions using the normal distribution and a continuity correction, while for continuous variables, a two-sample *t* test allowing for unequal variances was used. Unadjusted incidence rates per 1000 person-years were reported. As such, risk was standardized for variable follow-up periods within each cohort. The relative risk of new-onset cardiovascular events between the narcolepsy and non-narcolepsy cohorts was estimated using a multivariable Cox proportional hazards model adjusted for age, sex, region, insurance type, and relevant comorbidities (Supplementary Table S3 lists the comorbidities adjusted for in the analysis). Adjusted hazard ratios (HRs) and 95% confidence intervals (CIs) were reported. All patient characteristics were measured throughout the baseline period (the 6 months up to and including the cohort entry date), except for age, sex, U.S. geographic region, and insurance type, which were measured on the cohort entry date. A post hoc sensitivity analysis was performed by stratifying the patient cohort by narcolepsy subtype (NT1 and NT2).

Descriptive statistics were reported for outcomes in which the sample size was insufficient (*n* < 30 in either cohort) and/or the number of events was insufficient (<5 outcome events per covariate in the model). Analyses were performed using de-identified data without access to personally identifiable information. All statistical analyses were conducted using the Aetion Evidence Platform, which conducts statistical comparisons using R version 3.4.2 [29, 30].

## Results

### Study population

The MarketScan database included 54 239 110 patients who were 18 years or older and had at least 1 day of medical and pharmacy coverage during the study period. There were 12 836 patients with narcolepsy in the database, but for 10 of them, three matched non-narcolepsy control patients could not be identified. Additionally, 10 patients with narcolepsy and 37 non-narcolepsy control patients entered the cohort but their earliest day of insurance coverage discontinuation occurred on the first day of follow-up. Therefore, these patients were not included in any analysis. After applying all eligibility criteria, 12 816 and 38 441 patients were included in the narcolepsy and matched non-narcolepsy cohorts, respectively. In both cohorts, approximately 67% were female and 49% were from the southern United States, and the large majority (>96%) had commercial insurance (Table 1). Baseline demographics were similar between the cohorts.

**Table 1.** Baseline characteristics of patients diagnosed with narcolepsy and matched non-narcolepsy controls

Characteristic	Patients diagnosed with narcolepsy *n* = 12 816	Matched non-narcolepsy controls *n* = 38 441	*p*
Age, y, mean (SD)	38.1 (14.2)	38.5 (14.2)	.01
Sex, *n* (%)			
Male	4218 (32.9)	12 644 (32.9)	.98
Female	8598 (67.1)	25 797 (67.1)	.98
Region, *n* (%)			
Northeast	1692 (13.2)	5074 (13.2)	1.0
North Central	3426 (26.7)	10 273 (26.7)	.99
South	6283 (49.0)	18 849 (49.0)	.99
West	1289 (10.1)	3867 (10.1)	1.0
Unknown	126 (1.0)	378 (1.0)	1.0
Insurance type, *n* (%)			
Commercial	12 355 (96.4)	37 060 (96.4)	1.0
Medicare	461 (3.6)	1381 (3.6)	1.0

SD, standard deviation; y, years.

Patients included in the narcolepsy cohort had more comorbidities at baseline compared with matched non-narcolepsy controls (Table 2). The largest differences occurred in sleep apnea (33.8% and 2.2%), hypersomnia (31.7% and 0.2%), mood disorders (25.6% and 6.2%), anxiety disorders (21.7% and 7.2%), and headache/migraine (17.3% and 4.9%; all *p* < .001) for the narcolepsy cohort and non-narcolepsy cohort, respectively.

**Table 2.** Baseline comorbidities of patients diagnosed with narcolepsy and matched non-narcolepsy controls

Comorbidities, *n* (%)	Patients diagnosed with narcolepsy *n* = 12 816	Matched non-narcolepsy controls *n* = 38 441	Difference (95% CI) for non-narcolepsy controls − patients diagnosed with narcolepsy	*p*
Anxiety disorders	2780 (21.7)	2762 (7.2)	−14.5% (−15.3, −13.7)	<.001
Diabetes or diabetes/obesity medication	1406 (11.0)	3183 (8.3)	−2.7% (−3.3, −2.1)	<.001
Headache/Migraine	2212 (17.3)	1896 (4.9)	−12.3% (−13.0, −11.6)	<.001
Hyperlipidemia	1978 (15.4)	4084 (10.6)	−4.8% (−5.5, −4.1)	<.001
Hypersomnia	4067 (31.7)	88 (0.2)	−31.5% (−32.3, −30.7)	<.001
Mood disorders	3282 (25.6)	2368 (6.2)	−19.4% (−20.2, −18.7)	<.001
Periodic limb movement disorder	566 (4.4)	25 (0.1)	−4.4% (−4.7, −4.0)	<.001
Prior cardiovascular disease^a^	994 (7.8)	1547 (4.0)	−3.7% (−4.2, −3.2)	<.001
Pulmonary fibrosis or interstitial lung disease	34 (0.3)	35 (0.1)	−0.2% (−0.3, −0.1)	<0.01
Renal impairment	170 (1.3)	307 (0.8)	−0.5% (−0.8, −0.3)	<.01
Restless legs syndrome	647 (5.0)	102 (0.3)	−4.8% (−5.2, −4.4)	<.001
Sleep apnea	4328 (33.8)	843 (2.2)	−31.6% (−32.4, −30.7)	<.001

CI, confidence interval.

^a^For outcome analyses, patients were excluded if they had an event of interest in the 6-month period prior to study entry. Medical claim codes are in Supplementary Table S3.

### Risk of new-onset cardiovascular events

**Figure 2.**
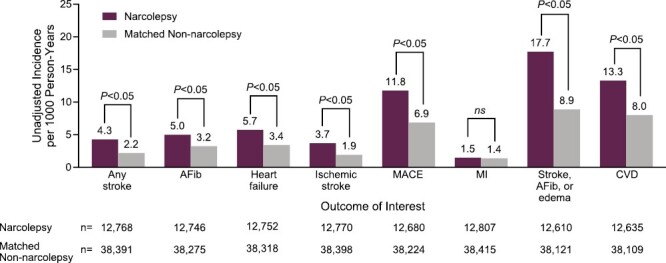
Unadjusted incidence rates for new-onset cardiovascular events in patients with narcolepsy and matched non-narcolepsy controls. AFib, atrial fibrillation; CVD, cardiovascular disease; MACE, major adverse cardiac event; MI, myocardial infarction; ns, *p* ≥ .05.

Crude incidence rates per 1000 person-years for outcomes were higher in patients with narcolepsy than in matched controls (Figure 2; all *p* < .05 except for myocardial infarction). The most common outcomes were composites: grouped instances of stroke, atrial fibrillation, or edema (17.7 and 8.9); CVD (13.3 and 8.0); and MACE (11.8 and 6.9) for the narcolepsy cohort and the non-narcolepsy cohort, respectively.

**Figure 3.**
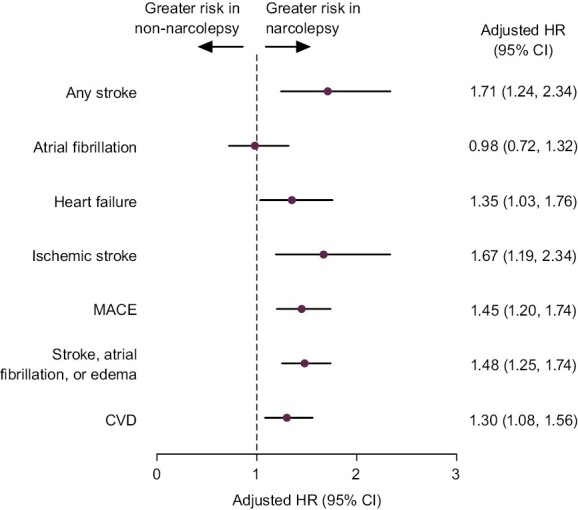
Adjusted HRs (95% CI) for incidence of new-onset cardiovascular events in patients with narcolepsy and matched non-narcolepsy controls. The relative risk of new-onset cardiovascular events between the narcolepsy and non-narcolepsy cohorts was estimated using a multivariable Cox proportional hazards model adjusted for age, sex, region, insurance type, and relevant comorbidities (Supplementary Table S3 lists the comorbidities adjusted for in the analysis). Adjusted HRs and 95% CIs are reported here. There were insufficient events to derive an HR for myocardial infarction. CI, confidence interval; CVD, cardiovascular disease; HR, hazard ratio; MACE, major adverse cardiac event.

Adjusted HRs demonstrated increased risk of new-onset cardiovascular events in the narcolepsy cohort compared with matched non-narcolepsy controls, including any stroke (adjusted HR [95% CI]: 1.71 [1.24, 2.34]); heart failure (1.35 [1.03, 1.76]); ischemic stroke (1.67 [1.19, 2.34]); MACE (1.45 [1.20, 1.74]); grouped instances of stroke, atrial fibrillation, or edema (1.48 [1.25, 1.74]); or CVD (1.30 [1.08, 1.56]; Figure 3). The HR could not be derived for the myocardial infarction outcome due to insufficient numbers of events.

### Sensitivity analysis

The majority of patients were considered to have NT2 (*n* = 9868; 77%) versus NT1 (*n* = 2948; 23%). Baseline characteristics and comorbidities of patients by subtype, and the matched controls, are reported in Tables 3–6. Baseline characteristics between patients with NT1 and their matched non-narcolepsy controls are shown in Table 3, and between patients with NT2 and their matched non-narcolepsy controls in Table 4. Patients included in the narcolepsy cohort with NT1 had more comorbidities at baseline compared with matched non-narcolepsy controls (Table 5). The largest differences occurred in sleep apnea (33.4% vs 2.1%), hypersomnia (28.8% vs 0.2%), mood disorders (26.6% vs 6.2%), anxiety disorders (22.7% vs 7.6%), and headache/migraine (17.3% vs 5.3%; all *p* < .001) for patients in the narcolepsy cohort with NT1 and non-narcolepsy cohort, respectively.

**Table 3.** Baseline characteristics of patients diagnosed with narcolepsy subtype 1 and matched non-narcolepsy controls (sensitivity analysis)

Characteristic	Patients diagnosed with narcolepsy subtype 1 *n* = 2948	Matched non-narcolepsy controls *n* = 8835	*p*
Age, y, mean (SD)	37.1 (14.2)	37.5 (14.2)	.22
Sex, *n* (%)			
Male	866 (29.4)	2591 (29.3)	.98
Female	2082 (70.6)	6244 (70.7)	.98
Region, *n* (%)			
Northeast	381 (12.9)	1141 (12.9)	1
North Central	771 (26.2)	2311 (26.2)	1
South	1428 (48.4)	4279 (48.4)	1
West	353 (12.0)	1059 (12.0)	1
Unknown	15 (0.5)	45 (0.5)	1
Insurance type, *n* (%)			
Commercial	2838 (96.3)	8505 (96.3)	1
Medicare	110 (3.7)	330 (3.7)	1

SD, standard deviation; y, years.

**Table 4.** Baseline characteristics of patients diagnosed with narcolepsy subtype 2 and matched non-narcolepsy controls (sensitivity analysis)

Characteristic	Patients diagnosed with narcolepsy subtype 2 *n* = 9868	Matched non-narcolepsy controls *n* = 29 606	*p*
Age, y, mean (SD)	38.4 (14.1)	38.7 (14.1)	.03
Sex, *n* (%)			
Male	3352 (34.0)	10 053 (34.0)	.99
Female	6516 (66.0)	19 553 (66.0)	.99
Region, *n* (%)			
Northeast	1311 (13.3)	3933 (13.3)	1
North Central	2655 (26.9)	7962 (26.9)	1
South	4855 (49.2)	14 570 (49.2)	1
West	936 (9.5)	2808 (9.5)	1
Unknown	111 (1.1)	333 (1.1)	1
Insurance type, *n* (%)			
Commercial	9517 (96.4)	28 555 (96.5)	1
Medicare	351 (3.6)	1051 (3.5)	1

SD, standard deviation; y, years.

**Table 5.** Baseline comorbidities of patients diagnosed with narcolepsy subtype 1 and matched non-narcolepsy controls (sensitivity analysis)

Comorbidities, *n* (%)	Patients diagnosed with narcolepsy subtype 1 *n* = 2948	Matched non-narcolepsy controls *n* = 8835	Difference (95% CI) for non-narcolepsy controls − patients diagnosed with narcolepsy subtype 1	*p*
Anxiety disorders	668 (22.7)	670 (7.6)	−15.1% (−16.7, −13.4)	<.001
Diabetes or diabetes/obesity medication	309 (10.5)	682 (7.7)	−2.8% (−4.0, −1.5)	<.01
Headache/Migraine	509 (17.3)	470 (5.3)	−11.9% (−13.4, −10.5)	<.001
Hyperlipidemia	427 (14.5)	867 (9.8)	−4.7% (−6.1, −3.2)	<.01
Hypersomnia	848 (28.8)	20 (0.2)	−28.5% (−30.2, −26.9)	<.001
Mood disorders	783 (26.6)	552 (6.2)	−20.3% (−22.0, −18.6)	<.001
Periodic limb movement disorder	135 (4.6)	4 (0.0)	−4.5% (−5.3, −3.8)	<.001
Prior cardiovascular disease^a^	255 (8.6)	341 (3.9)	−4.8% (−5.9, −3.7)	<.001
Pulmonary fibrosis or interstitial lung disease	8 (0.3)	5 (0.1)	−0.2% (−0.4, 0.0)	<.01
Renal impairment	39 (1.3)	64 (0.7)	−0.6% (−1.1, −0.1)	<.01
Restless legs syndrome	174 (5.9)	21 (0.2)	−5.7% (−6.5, −4.8)	<.001
Sleep apnea	986 (33.4)	185 (2.1)	−31.4% (−33.1, −29.6)	<.001

CI, confidence interval.

^a^For outcome analyses, patients were excluded if they had an event of interest in the 6-month period prior to study entry. Medical claim codes are in Supplementary Table S3.

**Table 6.** Baseline comorbidities of patients diagnosed with narcolepsy subtype 2 and matched non-narcolepsy controls (sensitivity analysis)

Comorbidities, *n* (%)	Patients diagnosed with narcolepsy subtype 2 *n* = 9868	Matched non-narcolepsy controls *n* = 29 606	Difference (95% CI) for non-narcolepsy controls − patients diagnosed with narcolepsy subtype 2	*p*
Anxiety disorders	2112 (21.4)	2092 (7.1)	−14.3% (−15.2, −13.5)	<.001
Diabetes or diabetes/obesity medication	1097 (11.1)	2501 (8.4)	−2.7% (−3.4, −2.0)	<.01
Headache/Migraine	1703 (17.3)	1426 (4.8)	−12.4% (−13.2, −11.7)	<.001
Hyperlipidemia	1551 (15.7)	3217 (10.9)	−4.9% (−5.7, −4.0)	<.001
Hypersomnia	3219 (32.6)	68 (0.2)	−32.4% (−33.3, −31.5)	<.001
Mood disorders	2499 (25.3)	1816 (6.1)	−19.2% (−20.1, −18.3)	<.001
Periodic limb movement disorder	431 (4.4)	21 (0.1)	−4.3% (−4.7, −3.9)	<.001
Prior cardiovascular disease^a^	739 (7.5)	1206 (4.1)	−3.4% (−4.0, −2.8)	<.001
Pulmonary fibrosis or interstitial lung disease	26 (0.3)	30 (0.1)	−0.2% (−0.3, −0.0)	<.01
Renal impairment	131 (1.3)	243 (0.8)	−0.5% (−0.8, −0.3)	<.01
Restless legs syndrome	473 (4.8)	81 (0.3)	−4.5% (−5.0, −4.1)	<.001
Sleep apnea	3342 (33.9)	658 (2.2)	−31.6% (−32.6, −30.7)	<.001

CI, confidence interval.

^a^For outcome analyses, patients were excluded if they had an event of interest in the 6-month period prior to study entry. Medical claim codes are in Supplementary Table S3.

Patients included in the narcolepsy cohort with NT2 had more comorbidities at baseline compared with matched non-narcolepsy controls (Table 6). The largest differences occurred in hypersomnia (32.6% vs 0.2%), sleep apnea (33.9% vs 2.2%), mood disorders (25.3% vs 6.1%), anxiety disorders (21.4% vs 7.1%), and headache/migraine (17.3% vs 4.8%; all *p* < .001) for patients in the narcolepsy cohort with NT2 and non-narcolepsy cohort, respectively.

**Figure 4.**
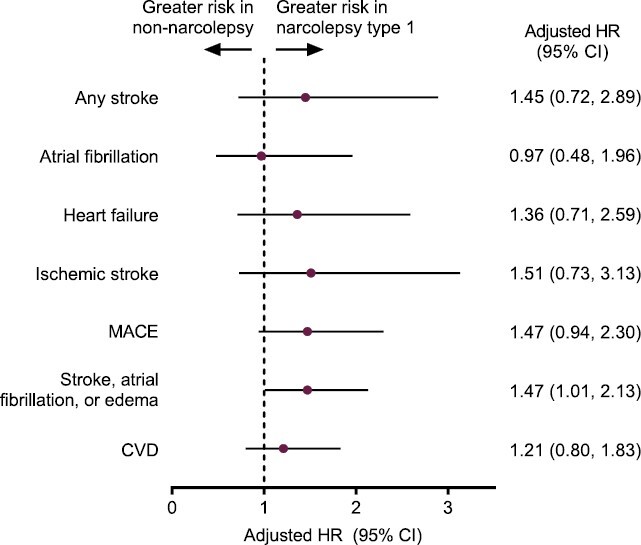

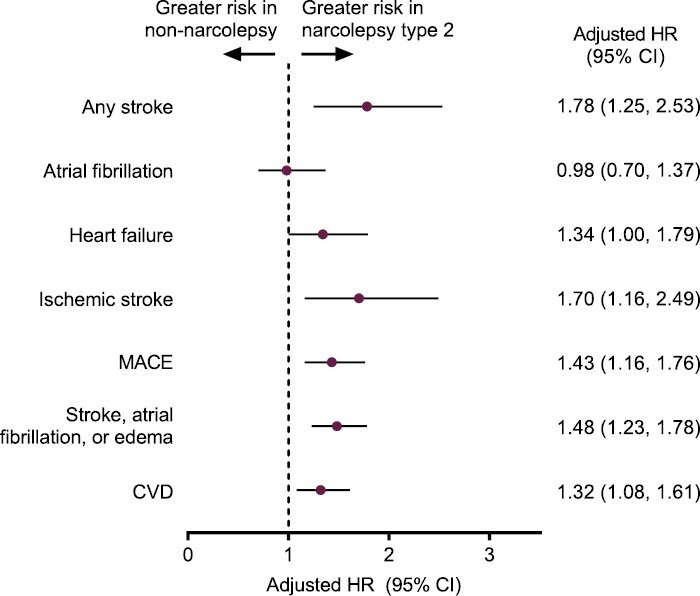
Sensitivity analysis: adjusted HRs (95% CI) for incidence of new-onset cardiovascular events in patients with (A) narcolepsy subtype 1 versus matched controls and (B) narcolepsy subtype 2 versus matched controls. CI, confidence interval; CVD, cardiovascular disease; HR, hazard ratio; MACE, major adverse cardiac event.

Among patients with NT1, adjusted HRs only demonstrated increased risk of grouped instances of stroke, atrial fibrillation, or edema in the narcolepsy cohort compared with matched non-narcolepsy controls (adjusted HR [95% CI]: 1.47 [1.01, 2.13]; Figure 4, A). Among patients with NT2, adjusted HRs demonstrated increased risk of new-onset cardiovascular events compared with matched non-narcolepsy controls, including any stroke (1.78 [1.25, 2.53]); heart failure (1.34 [1.00, 1.79]); ischemic stroke (1.70 [1.16, 2.49]); MACE (1.43 [1.16, 1.76]); grouped instances of stroke, atrial fibrillation, or edema (1.48 [1.23, 1.78]); or CVD (1.32 [1.08, 1.61]; Figure 4, B). The HR could not be derived for the myocardial infarction outcome due to insufficient numbers of events in the sensitivity analysis.

## Discussion

Earlier observational research characterizing medical comorbidities in patients with narcolepsy reported significantly greater odds of being diagnosed with cardiovascular comorbidities compared with non-narcolepsy controls [22]. Whereas previous efforts aimed to determine the cross-sectional prevalence of cardiovascular comorbidities in people with narcolepsy, CV-BOND endeavored to corroborate and build upon earlier findings by quantifying the longitudinal excess risk of new-onset cardiovascular events. The current study focuses solely on the development of new cardiovascular events following the diagnosis of narcolepsy. By ensuring that the exposure (narcolepsy) preceded the outcome (cardiovascular event), we satisfy one of the key criteria for assessing causality in epidemiology: temporality.

The resulting findings confirm and expand upon existing literature by demonstrating that the incidence rates of multiple cardiovascular events, including stroke, heart failure, ischemic stroke, MACE, grouped instances of stroke, atrial fibrillation, or edema, and CVD, were significantly higher in people with narcolepsy than matched non-narcolepsy controls. After controlling for differences in demographics, comorbidities, and other factors that could have affected cardiovascular risk, increased risk of cardiovascular events persisted for most outcomes. The exception was atrial fibrillation, which has been associated with other comorbidities [31–36] that were controlled for within the Cox model. Persistent increased risk for the onset of cardiovascular events provides evidence of temporal relationships between narcolepsy and poor cardiovascular outcomes. These trends were observed regardless of narcolepsy subtype. Hence, despite pathophysiologic differences between narcolepsy subtypes 1 and 2, the observed increased risk in the primary analysis is not being driven by one narcolepsy subtype. For clinicians, this highlights the importance of considering any patient with narcolepsy to be at increased risk for future cardiovascular events.

Extensive prior research supports the biological plausibility of narcolepsy as a determinant of elevated cardiovascular risk [8]. Destabilization of the sleep-wake cycle affects normal oscillations in blood pressure and attenuates the nocturnal fall in blood pressure, a phenomenon known to increase the risk of CV events [37]. In healthy patients, nocturnal blood pressure dipping occurs when blood pressure decreases >10% to 20% at night. Blunted nocturnal dipping occurs when blood pressure fails to achieve optimal levels of reduction, defined as ≤10% reduction during nighttime sleep. Patients with narcolepsy are far more likely to exhibit the non-dipping blood pressure phenotype [24]. As a result, risk of cardiovascular mortality is significantly (*p* < .003) increased in people who do not exhibit nocturnal blood pressure dipping [38].

The underlying pathophysiology of narcolepsy may offer additional insight on the potential mechanisms mediating a heightened risk of cardiovascular events. Hypocretins, a pair of excitatory neuropeptides (hypocretin-1 and hypocretin-2) that are responsible for stabilization of sleep and wake regulation [39, 40], are also involved in the modulation of blood pressure and heart rate during sleep/wake states. Their role is essential for normal nocturnal blood pressure dipping, which can become dysregulated when hypocretin is deficient [37]. Hypocretin deficiency is a core feature of NT1, which is characterized by autoimmune destruction of hypocretin neurons in the hypothalamus and reduced CSF hypocretin signaling [41–43]. Although the pathophysiology of NT2 is less clear, moderate hypocretin neuronal loss or insufficient release of hypocretin may still be present in many patients with NT2 [1, 41, 43, 44]. Hypocretins regulate several other central and peripheral processes, such as insulin secretion, resulting in metabolic dysfunction when levels are low [8, 37, 45–48]. Chronic dysregulation of hypocretin-related metabolic effects may be linked to the development of disorders known to negatively impact healthy cardiovascular function, such as obesity, diabetes, and dyslipidemia [8, 49–51]. Despite a possible role for this mechanism in patients with NT1, hypocretin deficiency may not fully explain the increased risk for cardiovascular events. A relatively small number of patients with NT1 was available for analysis, resulting in wider CIs for the NT1 group than for the NT2 group. This smaller sample size may have resulted in a potentially underpowered NT1–cardiovascular risk relationship.

Through a confluence of these and other factors, narcolepsy is associated with an excess prevalence of comorbid conditions known to confer increased cardiovascular risk. Rates of hypertension, diabetes, and dyslipidemia are significantly higher in patients with narcolepsy than in age- and gender-matched controls [21–23]. Obesity, a well-known risk factor for developing CVD, has been frequently associated with narcolepsy [21–23, 52]. This pattern of increased cardiovascular and cardiometabolic burden is consistent with evidence from the current study. The proximate origin of these comorbidities in narcolepsy may be a combination of altered metabolism, changes in eating patterns, and decreased physical activity [53]. Weight gain may be attributed to changes in the basal metabolic rate and changes in eating behaviors, such as increased appetite and high-calorie food consumption, associated with EDS [53, 54]. Children with NT1 have been shown to be at increased risk for developing obesity, with weight gain starting at disease onset [4, 55, 56]. As CVD is the leading cause of death in the U.S. general population [57], increased risk of cardiovascular events in narcolepsy may explain its association with a 1.5-fold increase in all-cause mortality [58]. Increased mortality rates are feasibly implicated from the complications of the condition itself, comorbid conditions associated with a narcolepsy diagnosis that carry their own mortality risk, or from a combination of narcolepsy and the comorbid medical conditions [58].

The interrelationship between sleep and cardiovascular health is well established, including disorders of sleep regulation and sleep-related breathing disorders, such as obstructive sleep apnea (OSA) [8]. Indeed, OSA is historically recognized by clinicians and guideline developers, including the American Heart Association, as an important risk factor for CVD [59]. OSA is prevalent among patients who have hypertension, atrial fibrillation and other arrhythmias, heart failure, coronary artery disease, cerebrovascular disease and stroke, and pulmonary hypertension [59]. Additionally, patients with OSA have increased rates of new-onset cardiovascular events, as well as all-cause mortality, compared with matched controls who do not have OSA [60–66]. Similar associations between narcolepsy and CVD have been observed [22, 23]. The associations were directionally similar and comparable in magnitude to OSA, despite differences across studies in terms of study designs, population characteristics, analytic methods, and study durations [22, 23]. Evidence shows that both OSA and narcolepsy are associated with increased risk of incident MACE. A meta-analysis found that the risk of new-onset MACE was higher with OSA that was moderate (RR, 1.16) or severe (RR, 2.04), compared with controls [66]. In CV-BOND, the risk of new-onset MACE also was higher with narcolepsy compared with non-narcolepsy controls (adjusted HR, 1.45). Thus, although the pathophysiology of narcolepsy is profoundly different from that of OSA, it is evidently similarly associated with cardiovascular dysfunction and risk.

Despite the relationship between OSA with CVD and elevated risk of new cardiovascular events, OSA often goes undiagnosed and untreated among patients receiving cardiovascular care [59]. Recognizing this unmet need, the AASM recently established an initiative to partner with other specialties, particularly cardiology, to educate clinicians who offer care for patients with sleep disorders. Although the AASM has emphasized the collaboration on OSA [67], findings of the current study, in conjunction with existing epidemiological literature, offer compelling reasons to broaden the focus to include narcolepsy as a priority. The totality of evidence suggests that educational initiatives about narcolepsy and its implications for cardiovascular health are warranted. The goal of education would be to make clinicians aware that the cardiovascular risk of narcolepsy, like OSA, should be given substantial weight to improve disease surveillance and develop comprehensive treatment plans for patients that consider both their present and future cardiovascular health.

Strengths of CV-BOND include a large total sample size, which permitted rigorous statistical analysis of individual cardiovascular events. Second, confounding was minimized by matching patients with narcolepsy with a non-narcolepsy control cohort on multiple characteristics and risk factors, and then using a multivariable Cox model to further adjust for covariates. Third, the narcolepsy cohort in the MarketScan database appears representative of the broader U.S. narcolepsy population in terms of baseline comorbidities (e.g. psychiatric disorders, sleep disorders, and cardiometabolic conditions) [21–23, 68]. About two-thirds (67%) were female, consistent with prior evidence that narcolepsy may be more common in women than in men [21]. However, this may be related to a greater tendency of women to seek medical attention compared with men [69].

Limitations include those common to insurance claims database studies. Possible misclassification bias of narcolepsy was mitigated by requiring two claims within 6 months for assignment to the narcolepsy cohort. Consequently, the study population could have had more severe and/or poorly controlled narcolepsy symptoms, and thus possibly higher rates of new-onset cardiovascular events, than the U.S. narcolepsy population. Additionally, findings may not be generalizable to uninsured people or those outside the United States. Claims that occurred more than 6 months before cohort entry were not included in the analysis, potentially overestimating incidence rates by including prevalent conditions. Variables such as disease duration, hypocretin levels, and body weight may increase cardiovascular risk for individuals with narcolepsy, but could not be controlled for in the model. Additionally, narcolepsy treatment may likewise be implicated in the observed results. Further research to explore the effect of medications indicated for narcolepsy may further isolate which individuals with narcolepsy are at increased risk for new-onset cardiovascular conditions, and why.

In conclusion, these results build on the current understanding of the association between cardiovascular health and narcolepsy, with the novel finding that new-onset cardiovascular events occur at a higher rate in people with narcolepsy compared with matched non-narcolepsy controls. CV-BOND utilized a robust design that permitted observation of purely new-onset cardiovascular events by excluding patients with a history of the outcome of interest. The observed phenomenon of an increased incidence of cardiovascular events was not due to any imbalance of measured confounders, as adjustment for those factors in CV-BOND did not negate the significance of our findings. This line of evidence suggests that the clinical profile of patients with narcolepsy is dynamic, evolving over time, placing a progressively greater burden on patients, and accelerating their risk of poor cardiovascular outcomes in the future. Patients who do not present early on with CVD/cardiometabolic disease may still accumulate cardiovascular risk at a greater rate over time due to the biological consequences associated with narcolepsy. It remains unknown why patients with narcolepsy are at an increased risk of new-onset cardiovascular events compared with individuals without narcolepsy. Although adjustments were made for known comorbidities in this study, the extent to which the disease itself, duration of the disease, and the dosage, type, and duration of treatment affect the relationship between narcolepsy and cardiovascular risk remains unclear. More collaboration is needed between researchers in sleep medicine and cardiology to continue evaluating the biological mechanisms resulting in increased cardiovascular and cardiometabolic risk in patients with narcolepsy. In addition, clinicians must consider not only the present clinical state of their patients with narcolepsy, but how risk for poor cardiovascular outcomes accumulates over their lifetime when considering treatment options. Appreciation and understanding of how the biology of narcolepsy and its associated comorbidities impact cardiovascular risk is essential for optimizing patient-centric treatment. Appropriate treatment decisions should include an informed discussion of therapies with proven effectiveness for narcolepsy symptoms and safety profiles that do not further jeopardize the current or future health of patients.

## Supplementary Material

zsad161_suppl_Supplementary_Material

## Data Availability

All relevant data are provided within the manuscript and supporting files.
