# Supplementary material for: Cardiovascular Burden of Narcolepsy Disease (CV-BOND): a real-world evidence study
Source: Sleep. 2023 Jun 12;46(10):zsad161. doi: 10.1093/sleep/zsad161 (PMC10566243; doi:10.1093/sleep/zsad161)
Supplement: zsad161_suppl_Supplementary_Material [file zsad161_suppl_supplementary_material.docx]

# SUPPLEMENTAL MATERIAL FOR:

# Cardiovascular Burden of Narcolepsy Disease (CV-BOND): A Real-World Evidence Study

Rami H. Ben-Joseph^1,^*; Ragy Saad^1,^*; Jed Black^1,2^; Elizabeth C. Dabrowski^3^;
Ben Taylor^3^; Sophia Gallucci^3^; Virend K. Somers^4^

^1^Jazz Pharmaceuticals, Palo Alto, CA, USA; ^2^Stanford University Center for Sleep Sciences and Medicine, Palo Alto, CA, USA; ^3^Aetion, Inc., New York, NY, USA; ^4^Department of Cardiovascular Medicine, Mayo Clinic, Rochester, MN, USA

**Corresponding author:**

Rami H. Ben-Joseph

Former head, Big Data Real World Evidence

Jazz Pharmaceuticals

3170 Porter Drive

Palo Alto, CA 94304

Phone: (858) 900-1309

Email: [Ramitali@yahoo.com](mailto:Ramitali@yahoo.com)

# *Employed by Jazz Pharmaceuticals at the time this research was conducted.

**Supplemental Contents**

Table S1. Diagnostic testing codes for narcolepsy claims

Table S2. Definitions of outcomes analyzed in this study

Table S3. Definitions of comorbidities

## Table S1. Diagnostic testing codes for narcolepsy claims

| **Test** | **Definition** |
| --- | --- |
| Multiple sleep latency test or polysomnography test | CPT: 95805 |
| Polysomnography | CPT: 95782, 95783, 95807, 95808, 95809, 95810, 95811 |
| Routine venipuncture for collection of specimens(s), single home bound, nursing home, or skilled nursing facility patient | CPT: S9529 |
| Routine venipuncture for collection of specimens | CPT: G0001 |
| Specimen collection: phlebotomy | CPT: 36400-36425 |
| Radiology | CPT: 70010-76999 |
| Nuclear radiology | CPT: 78000-78799 |
| Pathology and laboratory | CPT: 80000-89999 |
| Home sleep apnea test | CPT: 95800, 95801, 95806  HCPCS: G0398, G0399, G0400 |

CPT, Current Procedural Terminology; HCPCS, Healthcare Common Procedure Coding System.

Reference: Carls G, Reddy SR, Broder MS, et al. Burden of disease in pediatric narcolepsy: a claims-based analysis of health care utilization, costs, and comorbidities. *Sleep Med*. 2020;66:110-118. doi: 10.1016/j.sleep.2019.08.008.

## Table S2. Definitions of outcomes analyzed in this study

| **Outcome** | **Definition** |
| --- | --- |
| Any stroke | An inpatient or outpatient medical claim with 1 of the following diagnoses in the primary position:  ICD-9-CM: 430, 431, 434, 436  ICD-10-CM: I60, I61, I63, I64  Reference: McCormick N, Bhole V, Lacaille D, Avina-Zubieta JA. Validity of diagnostic codes for acute stroke in administrative databases: a systematic review. *PLoS One.* 2015;10(8):e0135834. doi: 10.1371/journal.pone.0135834. |
| Atrial fibrillation | An inpatient or outpatient medical claim with 1 of the following diagnoses in the primary position:  ICD-9-CM: 427.31, 427.32  ICD-10-CM: I48  Reference: Jensen PN, Johnson K, Floyd J, Heckbert SR, Carnahan R, Dublin S. A systematic review of validated methods for identifying atrial fibrillation using administrative data. *Pharmacoepidemiol Drug Saf*. 2012;21(suppl 1[0 1]):141-147. doi: 10.1002/pds.2317. |
| Heart failure | An inpatient or outpatient medical claim with 1 of the following diagnoses in the primary position:  ICD-9-CM: 428  ICD-10-CM: I09.9, I11.0, I13.0, I13.2, I25.5, I42.0, I42.5, I42.6, I42.7, I42.8, I42.9, I43, I50, P29.0  Reference: So L, Evans D, Quan H. ICD-10 coding algorithms for defining comorbidities of acute myocardial infarction. *BMC Health Serv Res*. 2006;6:161. doi: 10.1186/1472-6963-6-161. |
| Ischemic stroke | An inpatient or outpatient medical claim with 1 of the following diagnoses in the primary position:  ICD-9-CM: 434, 436  ICD-10-CM: I63, I64  Reference: McCormick N, Bhole V, Lacaille D, Avina-Zubieta JA. Validity of diagnostic codes for acute stroke in administrative databases: a systematic review. *PLoS One*. 2015;10(8):e0135834. doi: 10.1371/journal.pone.0135834. |
| MACE | An inpatient or outpatient medical claim with 1 of the following diagnoses or procedures in the primary position:  Myocardial infarction  ICD-9-CM: 410  ICD-10-CM: I21, I22  Ischemic stroke  ICD-9-CM: 434, 436  ICD-10-CM: I63, I64  Heart failure  ICD-9-CM: 428  ICD-10-CM: I09.9, I11.0, I13.0, I13.2, I25.5, I42.0, I42.5, I42.6, I42.7, I42.8, I42.9, I43, I50, P29.0  Acute coronary syndrome  ICD-9-CM: 410, 411  ICD-10-CM: I20.0, I21.09, I21.11, I21.19, I21.29, I21.3, I21.4, I24.0  Coronary artery bypass graft  CPT/HCPCS: 00566, 00567, 33510, 33511, 33512, 33513, 33514, 33516, 33517, 33518, 33519, 33520, 33521, 33522, 33523, 33525, 33528, 33530, 33533, 33534, 33535, 33536, 35600, 4110F, 75762, 75764, 75766, 75767, 93551, C9604, C9605, G8158, G8159, G8160, G8161, G8162, G8163, G8164, G8165, G8166, G8167, G8170, G8171, G8172, G8497, G8544, G8573, G8574  ICD-10-CM: I25.70, I25.700, I25.701, I25.708, I25.71, I25.710, I25.711, I25.718, I25.719, I25.72, I25.720, I25.721, I25.728, I25.729, I25.73, I25.730, I25.731, I25.738, I25.739, I25.79, I25.790, I25.791, I25.798, I25.799, I25.810, T82.211, T82.211A, T82.211D, T82.211S, T82.212, T82.212A, T82.212D, T82.212S, T82.213, T82.213A, T82.213D, T82.213S, T82.218, T82.218A, T82.218D, T82.218S, Z95.1  ICD-10-PCS: 0210093, 0210098, 0210099, 021009C, 021009F, 021009W, 02100A3, 02100A8, 02100A9, 02100AC, 02100AF, 02100AW, 02100J3, 02100J8, 02100J9, 02100JC, 02100JF, 02100JW, 02100K3, 02100K8, 02100K9, 02100KC, 02100KF, 02100KW, 02100Z3, 02100Z8, 02100Z9, 02100ZC, 02100ZF, 0210344, 02103D4, 0210444, 0210493, 0210499, 0210499, 021049F, 021049W, 02104A3, 02104A8, 02104A9, 02104AC, 02104AF, 02104AW, 02104D4, 02104J3, 02104J8, 02104J9, 02104JC, 02104JF, 02104JW, 02104K3, 02104K8, 02104K9, 02104KC, 02104KF, 02104KW, 02104Z3, 02104Z8, 02104Z9, 02104ZC, 02104ZF, 0211093, 0211098, 0211099, 021109C, 021109F, 021109W, 02110A3, 02110A8, 02110A9, 02110AC, 02110AF, 02110AW, 02110J3, 02110J8, 02110J9, 02110JC, 02110JF, 02110JW, 02110K3, 02110K8, 02110K9, 02110KC, 02110KF, 02110KW, 02110Z3, 02110Z8, 02110Z9, 02110ZC, 02110ZF, 0211344, 02113D4, 0211444, 0211493, 0211498, 0211499, 021149C, 021149F, 021149W, 02114A3, 02114A8, 02114A9, 02114AC, 02114AF, 02114AW, 02114D4, 02114J3, 02114J8, 02114J9, 02114JC, 02114JF, 02114JW, 02114K3, 02114K8, 02114K9, 02114KC, 02114KF, 02114KW, 02114Z3, 02114Z8, 02114Z9, 02114ZC, 02114ZF, 0212093, 0212098, 0212099, 021209C, 021209F, 021209W, 02120A3, 02120A8, 02120A9, 02120AC, 02120AF, 02120AW, 02120J3, 02120J8, 02120J9, 02120JC, 02120JF, 02120JW, 02120K3, 02120K8, 02120K9, 02120KC, 02120KF, 02120KW, 02120Z3, 02120Z8, 02120Z9, 02120ZC, 02120ZF, 0212344, 02123D4, 0212444, 0212493, 0212498, 0212499, 021249C, 021249F, 021249W, 02124A3, 02124A8, 02124A9, 02124AC, 02124AF, 02124AW, 02124D4, 02124J3, 02124J8, 02124J9, 02124JC, 02124JF, 02124JW, 02124K3, 02124K8, 02124K9, 02124KC, 02124KF, 02124KW, 02124Z3, 02124Z8, 02124Z9, 02124ZC, 02124ZF, 0213093, 0213098, 0213099, 021309C, 021309F, 021309W, 02130A3, 02130A8, 02130A9, 02130AC, 02130AF, 02130AW, 02130J3, 02130J8, 02130J9, 02130JC, 02130JF, 02130JW, 02130K3, 02130K8, 02130K9, 02130KC, 02130KF, 02130KW, 02130Z3, 02130Z8, 02130Z9, 02130ZC, 02130ZF, 0213344, 02133D4, 0213444, 0213493, 0213498, 0213499, 021349C, 021349F, 021349W, 02134A3, 02134A8, 02134A9, 02134AC, 02134AF, 02134AW, 02134D4, 02134J3, 02134J8, 02134J9, 02134JC, 02134JF, 02134JW, 02134K3, 02134K8, 02134K9, 02134KC, 02134KF, 02134KW, 02134Z3, 02134Z8, 02134Z9, 02134ZC, 02134ZF, 0210498, B2020ZZ, B2021ZZ, B202YZZ, B2030ZZ, B2031ZZ, B203YZZ, B212010, B2120ZZ, B212110, B2121ZZ, B212Y10, B212YZZ, B213010, B2130ZZ, B213110, B2131ZZ, B213Y10, B213YZZ, B22300Z, B2230ZZ, B22310Z, B2231ZZ, B223Y0Z, B223YZZ, B223Z2Z, B223ZZZ, B233Y0Z, B233YZZ, B233ZZZ  Percutaneous coronary intervention  CPT/HCPCS: 92920, 92921, 92924, 92925, 92928, 92929, 92933, 92934, 92937, 92938, 92941, 92943, 92944, C9600, C9601, C9602, C9603, C9604, C9605, C9606, C9607, C9608  ICD-10-PCS: 02700D6, 02700DZ, 02700T6, 02700TZ, 02700Z6, 02700ZZ, 02703D6, 02703DZ, 02703T6, 02703TZ, 02703Z6, 02703ZZ, 02704D6, 02704DZ, 02704T6, 02704TZ, 02704Z6, 02704ZZ, 02710D6, 02710DZ, 02710T6, 02710TZ, 02710Z6, 02710ZZ, 02713D6, 02713DZ, 02713T6, 02713TZ, 02713Z6, 02713ZZ, 02714D6, 02714DZ, 02714T6, 02714TZ, 02714Z6, 02714ZZ, 02720D6, 02720DZ, 02720T6, 02720TZ, 02720Z6, 02720ZZ, 02723D6, 02723DZ, 02723T6, 02723TZ, 02723Z6, 02723ZZ, 02724D6, 02724DZ, 02724T6, 02724TZ, 02724Z6, 02724ZZ, 02730D6, 02730DZ, 02730T6, 02730TZ, 02730Z6, 02730ZZ, 02733D6, 02733DZ, 02733T6, 02733TZ, 02733Z6, 02733ZZ, 02734D6, 02734DZ, 02734T6, 02734TZ, 02734Z6, 02734ZZ, 0270046, 027004Z, 0270346, 027034Z, 0270446, 027044Z, 0271046, 027104Z, 0271346, 027134Z, 0271446, 027144Z, 0272046, 027204Z, 0272346, 027234Z, 0272446, 027244Z, 0273046, 027304Z, 0273346, 027334Z, 0273446, 027344Z  Reference: Miao B, Hernandez AV, Alberts MJ, Mangiafico N, Roman YM, Coleman CI. Incidence and predictors of major adverse cardiovascular events in patients with established atherosclerotic disease or multiple risk factors. *J Am Heart Assoc*. 2020;9(2):e014402. doi: 10.1161/JAHA.119.014402. |
| Myocardial infarction | An inpatient or outpatient medical claim with 1 of the following diagnoses in the primary position:  ICD-9-CM: 410  ICD-10-CM: I21, I22  Reference: McCormick N, Lacaille D, Bhole V, Avina-Zubieta JA. Validity of myocardial infarction diagnoses in administrative databases: a systematic review. *PLoS One*. 2014;9(3):e92286. doi: 10.1371/journal.pone.0092286. |
| Cardiovascular disease | Grouped instances of any stroke, atrial fibrillation, heart failure, and myocardial infarction (as defined above) |

CPT, Current Procedural Terminology; HCPCS, Healthcare Common Procedure Coding System; ICD-9, *International Classification of Diseases, Ninth Revision, Clinical Modification*; ICD-10, *International Classification of Diseases, Tenth Revision, Clinical Modification*; MACE, major adverse cardiac event.

## Table S3. Definitions of comorbidities

| **Outcome** | **Definition** |
| --- | --- |
| Anxiety disorders | An inpatient or outpatient medical claim with a diagnoses (in any position) from the CCS category 651  Reference: Black J, Reaven NL, Funk SE, et al. Medical comorbidity in narcolepsy: findings from the Burden of Narcolepsy Disease (BOND) study. *Sleep Med.* 2016;33:13-18. doi: 10.1016/j.sleep.2016.04.004. |
| Coronary revascularization | An inpatient or outpatient medical claim with a code for percutaneous coronary intervention or coronary artery bypass graft in any position.  Percutaneous coronary intervention:  CPT/HCPCS: 92980, 92981, 92982, 92973, 92984, 92995, 92996, G0290, G0291  ICD-9-CM procedure codes: 36.0, 00.66  ICD-10-CM procedure codes: 0270346, 027034Z, 02703D6, 02703DZ, 02703Z6, 02703ZZ, 0270446, 027044Z, 02704D6, 02704DZ, 02704Z6, 02704ZZ, 0271346, 027134Z, 02713D6, 02713DZ, 02713Z6, 02713ZZ, 0271446, 027144Z, 02714D6, 02714DZ, 02714Z6, 02714ZZ, 02723D6, 02723DZ, 02723Z6, 02723ZZ, 0272446, 027244Z, 02724D6, 02724DZ, 02724Z6, 02724ZZ, 0273346, 027334Z, 02733D6, 02733DZ, 02733Z6, 02733ZZ, 0273446, 027344Z, 02734D6, 02734DZ, 02734Z6, 02734ZZ  Coronary artery bypass graft:  CPT/HCPCS: 33510, 33511, 33512, 33513, 33514, 33516, 33517, 33518, 33519, 33521, 33522, 33523, 33533, 33534, 33535, 33536  ICD-9-CM procedure codes: 36.1, 36.2, 36.31, 36.32  ICD-10-CM procedure codes: 0210093, 0210098, 0210099, 021009C, 021009F, 021009W, 02100A3, 02100A8, 02100A9, 02100AC, 02100AF, 02100AW, 02100J3, 02100J8, 02100J9, 02100JC, 02100JF, 02100JW, 02100K3, 02100K8, 02100K9, 02100KC, 02100KF, 02100KW, 02100Z3, 02100Z8, 02100Z9, 02100ZC, 02100ZF, 0211093, 0211098, 0211099, 021109C, 021109F, 021109W, 02110A3, 02110A8, 02110A9, 02110AC, 02110AF, 02110AW, 02110J3, 02110J8, 02110J9, 02110JC, 02110JF, 02110JW, 02110K3, 02110K8, 02110K9, 02110KC, 02110KF, 02110KW, 02110Z3, 02110Z8, 02110Z9, 02110ZC, 02110ZF, 0212093, 0212098, 0212099, 021209C, 021209F, 021209W, 02120A3, 02120A8, 02120A9, 02120AC, 02120AF, 02120AW, 02120J3, 02120J8, 02120J9, 02120JC, 02120JF, 02120JW, 02120K3, 02120K8, 02120K9, 02120KC, 02120KF, 02120KW, 02120Z3, 02120Z8, 02120Z9, 02120ZC, 02120ZF, 0213093, 0213098, 0213099, 021309C, 021309F, 021309W, 02130A3, 02130A8, 02130A9, 02130AC, 02130AF, 02130AW, 02130J3, 02130J8, 02130J9, 02130JC, 02130JF, 02130JW, 02130K3, 02130K8, 02130K9, 02130KC, 02130KF, 02130KW, 02130Z3, 02130Z8, 02130Z9, 02130ZC, 02130ZF, 021K0Z8, 021K0Z9, 021K0ZC, 021K0ZW, 021K4Z8, 021K4Z9, 021K4ZC, 021K4ZW, 021L0Z8, 021L0Z9, 021L0ZC, 021L4Z8, 021L4Z9, 021L4ZC, 021K0Z5, 021L0Z5, 021K4Z5, 021L4Z5  Reference: Shah BR, Cowper PA, O’Brien SM, et al. Association between physician billing and cardiac stress testing patterns following coronary revascularization. *JAMA*. 2011;306(18):1993-2000. doi: 10.1001/jama.2011.1604. |
| Depressive disorders | An inpatient or outpatient medical claim with 1 of the following diagnoses in the any position:  ICD-9-CM: 300.4, 301.12, 309.0, 309.1, 311  ICD-10-CM: F20.4, F31.3, F31.4, F31.5, F32, F33, F34.1, F41.2, F43.2  Reference: Quan H, Sundararajan V, Halfon P, et al. Coding algorithms for defining comorbidities in ICD-9-CM and ICD-10 administrative data. *Med Care*. 2005;43(11):1130-1139. doi: 10.1097/01.mlr.0000182534.19832.83. |
| Diabetes or diabetes/obesity medication | An inpatient or outpatient medical claim with a diagnoses (in any position) from the CCS categories 49, 50  OR  A drug claim with 1 of the following generic drug names: metformin, canagliflozin, dapagliflozin, ertugliflozin, empagliflozin, alogliptin, linagliptin, miglitol, pioglitazone, rosiglitazone, saxagliptin, sitagliptin, nateglinide, lixisenatide, glipizide, repaglinide, glyburide, glibenclamide, glimepiride, tolbutamide, tolazamide, chlorpropamide, gliclazide, acarbose, voglibose, vildagliptin, bromocriptine, teneligliptin, trelagliptin, anagliptin, exenatide, liraglutide, dulaglutide, semaglutide, albiglutide, pramlintide, insulin, orlistat, lorcaserin, phentermine/topiramate, naltrexone/bupropion, phentermine, phendimetrazine, diethylpropion  Reference: Black J, Reaven NL, Funk SE, et al. Medical comorbidity in narcolepsy: findings from the Burden of Narcolepsy Disease (BOND) study. *Sleep Med.* 2016;33:13-18. doi: 10.1016/j.sleep.2016.04.004. |
| Headache/ Migraine | An inpatient or outpatient medical claim with a diagnoses (in any position) from the CCS category 84  Reference: Black J, Reaven NL, Funk SE, et al. Medical comorbidity in narcolepsy: findings from the Burden of Narcolepsy Disease (BOND) study. *Sleep Med.* 2016;33:13-18. doi: 10.1016/j.sleep.2016.04.004. |
| Hyperlipidemia | An inpatient or outpatient medical claim with 1 of the following diagnoses in any position:  ICD-9-CM: 272.0, 272.1, 272.2, 272.3, 272.4  ICD-10-CM: E78  Reference: Southern DA, Norris CM, Quan H, et al. An administrative data merging solution for dealing with missing data in a clinical registry: adaptation from ICD-9 to ICD-10. *BMC Med Res Methodol*. 2008;8:1. doi: 10.1186/1471-2288-8-1. |
| Hypersomnia | An inpatient or outpatient medical claim with 1 of the following diagnoses in any position:  ICD-9-CM: 327.1, 780.54  ICD-10-CM: G47.1, F51.13, F51.19  Reference: Blumentals WA, Song X. The safety of oseltamivir in patients with influenza: analysis of healthcare claims data from six influenza seasons. *MedGenMed.* 2007;9(4):23. PMCID: PMC2234272. |
| Hyperuricemia | An inpatient or outpatient medical claim with the following diagnosis in any position:  ICD-10-CM: E79.0  Reference: Engel B, Gomm W, Broich K, Maier W, Weckbecker K, Haenisch B. Hyperuricemia and dementia—a case-control study. *BMC Neurology.* 2018;18(1):131. doi: 10.1186/s12883-018-1136-y. |
| Mood disorders | An inpatient or outpatient medical claim with a diagnosis (in any position) from the CCS category 657  Reference: Black J, Reaven NL, Funk SE, et al. Medical comorbidity in narcolepsy: findings from the Burden of Narcolepsy Disease (BOND) study. *Sleep Medicine.* 2016;33:13-18. doi: 10.1016/j.sleep.2016.04.004. |
| Periodic limb movement disorder | An inpatient or outpatient medical claim with 1 of the following diagnoses in any position:  ICD-9-CM: 327.51  ICD-10-CM: G47.61  Reference: Black J, Reaven NL, Funk SE, et al. Medical comorbidity in narcolepsy: findings from the Burden of Narcolepsy Disease (BOND) study. *Sleep Medicine.* 2016;33:13-18. doi: 10.1016/j.sleep.2016.04.004. |
| Prior cardiovascular disease | An inpatient or outpatient medical claim with 1 of the following diagnoses in any position:  ICD‐9‐CM: 410, 411, 412, 413, 414.0, 427.3, 427.4, 427.5, 428, V45, 430, 431, 432, 433, 434, 435, 436, 437, 438, 440, 441, 444, V12.5  ICD‐10‐CM: I20.0, I20.9, I21, I22, I25.1, I25.2, I46, I48, I49, I50, Z95, I60, I61, I62, I63, I64, G45, I70, I71, I72, I73, I74, I75, I76, I77, I78, I79  Reference: Nyström T, Bodegard J, Nathanson D, Thuresson M, Norhammar A, Eriksson JW. Second line initiation of insulin compared with DPP-4 inhibitors after metformin monotherapy is associated with increased risk of all-cause mortality, cardiovascular events, and severe hypoglycemia. *Diabetes Res Clin Pract*. 2017;123:199-208. doi: 10.1016/j.diabres.2016.12.004. |
| Pulmonary fibrosis or interstitial lung disease | An inpatient or outpatient medical claim with 1 of the following diagnoses in any position:  ICD-9-CM-CM: 515, 516.3, 516.30, 516.31, 516.32, 516.33, 516.34, 516.35, 516.36, 516.37, 516.8, 516.9  ICD-10-CM-CM: J84.09, J84.10, J84.111, J84.112, J84.113, J84.114, J84.115, J84.116, J84.117, J84.2, J84.89, J84.9  Reference: Jones N, Schneider G, Kachroo S, Rotella P, Avetisyan R, Reynolds MW. A systematic review of validated methods for identifying pulmonary fibrosis and interstitial lung disease using administrative and claims data. *Pharmacoepidemiol Drug Saf.* 2012;21:256-260. doi: 10.1002/pds.2338. |
| Rapid eye movement behavior disorder | An inpatient or outpatient medical claim with 1 of the following diagnoses in any position:  ICD-9-CM: 327.42  ICD-10-CM: G47.52  Reference: Black J, Reaven NL, Funk SE, et al. Medical comorbidity in narcolepsy: findings from the Burden of Narcolepsy Disease (BOND) study. *Sleep Med.* 2016;33:13-18. doi: 10.1016/j.sleep.2016.04.004. |
| Renal impairment | An inpatient or outpatient medical claim with 1 of the following diagnoses in any position:  ICD-9-CM: 403.11, 403.91, 404.12, 404.92, 585, 586, V42.0, V45.1, V56.0, V56.8, 584.8, 584.9  ICD-10-CM: I12.0, I13.1, N18, N19, N25.0, Z49.0–Z49.2, Z94.0, Z99.2, N17.8, N17.9  Reference: Quan H, Sundararajan V, Halfon P, et al. Coding algorithms for defining comorbidities in ICD-9-CM and ICD-10 administrative data. *Med Care*. 2005;43(11):1130-1139. doi: 10.1097/01.mlr.0000182534.19832.83.  With the addition of other ICD-10 codes identified by Dr. Somers. |
| Restless legs syndrome | An inpatient or outpatient medical claim with 1 of the following diagnoses in any position:  ICD-9-CM: 333.94  ICD-10-CM: G25.81  Reference: Black J, Reaven NL, Funk SE, et al. Medical comorbidity in narcolepsy: findings from the Burden of Narcolepsy Disease (BOND) study. *Sleep Med.* 2016;33:13-18. doi: 10.1016/j.sleep.2016.04.004. |
| Sleep apnea | An inpatient or outpatient medical claim with 1 of the following diagnoses in any position:  ICD-9-CM: 327.20, 327.21, 327.23, 327.27, 327.29  ICD-10-CM: G47.30, G47.31, G47.33, G47.37, G47.39  Reference: Black J, Reaven NL, Funk SE, et al. Medical comorbidity in narcolepsy: findings from the Burden of Narcolepsy Disease (BOND) study. *Sleep Med.* 2016;33:13-18. doi: 10.1016/j.sleep.2016.04.004. |
| Use of antihypertensives | A drug claim with 1 of the following generic drug names: fosinopril, moexipril, enalapril, perindopril, captopril, trandolapril, quinapril, ramipril, benazepril, lisinopril, azilsartan, eprosartan, candesartan, telmisartan, olmesartan, irbesartan, valsartan, losartan, prazosin, doxazosin, terazosin, acebutolol, atenolol, betaxolol, bisoprolol, carteolol, carvedilol, labetalol, metoprolol, nadolol, nebivolol, penbutolol, pindolol, propranolol, sotalol, timolol, esmolol, nitroglycerin, amlodipine, bepridil, clevidipine, diltiazem, felodipine, isradipine, nicardipine, nifedipine, nimodipine, nisoldipine, verapamil, pitavastatin, fluvastatin, rosuvastatin, pravastatin, lovastatin, atorvastatin, simvastatin, niacin, cholestyramine, colesevelam, colestipol, ezetimibe, fenofibrate, gemfibrozil, alirocumab, evolocumab, chlorothiazide, chlorthalidone, hydrochlorothiazide, indapamide, metolazone, bumetanide, ethacrynic acid, furosemide, torsemide, amiloride, eplerenone, spironolactone, triamterene  OR  A drug claim with the WHO ATC code prefix C02  Reference: None. |

ATC, Anatomical Therapeutic Chemical; CCS, Clinical Classification Software; CPT, Current Procedural Terminology; HCPCS, Healthcare Common Procedure Coding System; ICD-9, *International Classification of Diseases, Ninth Revision, Clinical Modification*; ICD-10, *International Classification of Diseases, Tenth Revision, Clinical Modification*; WHO, World Health Organization.
